# Classical homeopathy in the treatment of cancer patients - a prospective observational study of two independent cohorts

**DOI:** 10.1186/1471-2407-11-19

**Published:** 2011-01-17

**Authors:** Matthias Rostock, Johannes Naumann, Corina Guethlin, Lars Guenther, Hans H Bartsch, Harald Walach

**Affiliations:** 1 Tumour Biology Center at Albert Ludwig's University Freiburg, Germany; 2 Dept. of Evaluation Research in Complementary Medicine, University Hospital Freiburg, Germany; 3 Institute for Transcultural Health Studies and Samueli Institute, European Office, Europa Universität Viadrina, Frankfurt (Oder), Germany; 4 Institute of Complementary Medicine, University Hospital Zurich, Switzerland; 5 Institute for General Practice, Johann Wolfgang Goethe University Frankfurt, Germany

## Abstract

**Background:**

Many cancer patients seek homeopathy as a complementary therapy. It has rarely been studied systematically, whether homeopathic care is of benefit for cancer patients.

**Methods:**

We conducted a prospective observational study with cancer patients in two differently treated cohorts: one cohort with patients under complementary homeopathic treatment (HG; n = 259), and one cohort with conventionally treated cancer patients (CG; n = 380). For a direct comparison, matched pairs with patients of the same tumour entity and comparable prognosis were to be formed.

Main outcome parameter: change of quality of life (FACT-G, FACIT-Sp) after 3 months.

Secondary outcome parameters: change of quality of life (FACT-G, FACIT-Sp) after a year, as well as impairment by fatigue (MFI) and by anxiety and depression (HADS).

**Results:**

HG: FACT-G, or FACIT-Sp, respectively improved statistically significantly in the first three months, from 75.6 (SD 14.6) to 81.1 (SD 16.9), or from 32.1 (SD 8.2) to 34.9 (SD 8.32), respectively. After 12 months, a further increase to 84.1 (SD 15.5) or 35.2 (SD 8.6) was found. Fatigue (MFI) decreased; anxiety and depression (HADS) did not change.

CG: FACT-G remained constant in the first three months: 75.3 (SD 17.3) at t0, and 76.6 (SD 16.6) at t1. After 12 months, there was a slight increase to 78.9 (SD 18.1). FACIT-Sp scores improved significantly from t0 (31.0 - SD 8.9) to t1 (32.1 - SD 8.9) and declined again after a year (31.6 - SD 9.4). For fatigue, anxiety, and depression, no relevant changes were found.

120 patients of HG and 206 patients of CG met our criteria for matched-pairs selection. Due to large differences between the two patient populations, however, only 11 matched pairs could be formed. This is not sufficient for a comparative study.

**Conclusion:**

In our prospective study, we observed an improvement of quality of life as well as a tendency of fatigue symptoms to decrease in cancer patients under complementary homeopathic treatment. It would take considerably larger samples to find matched pairs suitable for comparison in order to establish a definite causal relation between these effects and homeopathic treatment.

## Background

Many cancer patients use complementary and alternative medicine (CAM) treatments. Homeopathy is one of the most popular CAM modalities for cancer patients in seven out of 14 European countries [1]. Homeopathy has traditionally been very popular in India and South America too, and is increasingly sought after also in the US [2].

Developed in the 18^th ^century by German physician Samuel Hahnemann, it is based on two principles, the Law of Similars ("similia similibus curentur: let likes be cured by likes") and Individualisation, and it makes use of a specific form of remedy preparation, the stepwise dilution and potentisation [3].

Homeopathy is discussed controversially as there is no plausible mode of action for the highly diluted remedies, and whether it is clinically effective is currently a matter of heated debate. While some reviews and meta-analyses find it potentially efficacious (e.g. [4], [5]), a recent analysis finds it no better than placebo [6]. However, the latter analysis has been heavily criticised and recently shown to be extremely dependent on decisions as to which trials to select for analysis [7]. Hence the debate is unresolved.

In cancer patients homeopathy has rarely been studied systematically. A Cochrane Review of homeopathic medicines for adverse effects of cancer treatments found eight randomised controlled studies with mixed results [8]. A second systematic review concluded that the "evidence is encouraging but not convincing" [9]. The effects of homeopathy on quality of life in cancer patients has been studied very rarely. Only two randomised trials used it as a secondary outcome, one with and one without positive results [10], [11]. A retrospective hypotheses generating study in a clinic specialising in the homeopathic care of cancer patients found that the majority of patients indicated that they had improved in QoL due to their homeopathic treatment, as well as in fatigue symptoms and psychological well-being (Rostock M, Hinrichs I, Walach H.: Homeopathic treatment of cancer patients: a retrospective analysis, submitted).

Most trials of homeopathy have not studied classical homeopathy that individualises treatments for patients, but used either fixed combinations for certain symptom clusters, or isopathy, i.e. the same substance that triggers an allergic response, or simplified versions of homeopathy. In those cases it is comparatively easy to conduct randomised, placebo controlled studies. We wanted to study the clinical effects of classical homeopathy. This entails complex interviews, selection of important symptoms with multiple cycles of adjustments according to feedback, and long term observations [12,13]. Blinding such procedures, although performed sometimes [14], is only possible for a short period, and there are grave doubts as to the validity of the results achieved by it. Patients with cancer or other serious chronic diseases who seek out complementary care normally have very clear preferences [15]. They are mostly unwilling to enter an experiment and submit to randomisation [16-20]. In the spirit of a staged evaluation approach it is mandatory to study the effects of treatments for patients who have actively chosen them, since free choice is part and parcel of a potentially important therapeutic step [21]. We therefore set out to study classical homeopathic care for cancer patients, as chosen by patients, including all elements of case taking, setting, social support and the dispensation of homeopathic remedies, and compare it with a conventional setting. We wanted to see whether patients benefit, overall, in QoL from homeopathic care. Therefore we conducted an observational study with two natural cohorts to monitor the developing of QoL under homeopathic and under conventional care. For a direct comparison we planned to form matched pairs out of patients with matchable case histories from both cohorts as a nested feasibility study.

## Methods

Over a period of 30 months all new patients who chose treatment either in two clinics specialising in homeopathic care (Clinica Santa Croce, Orselina, Switzerland, and Homeopathic Centre Oberland-Klinik, Weilheim, Germany) or in two conventional specialised oncological outpatient clinics with cancer care according to state of the art (Clinic for Interdisciplinary Oncology and Hematology, Freiburg, Germany, and Clinic for Oncology and Hematology, Offenburg, Germany) were approached and included in a prospective observational study, once they had given informed consent. All patients received the normal standard of care offered in each place without any experimental intervention or interference with the treatment plan. The homeopathic clinics offered a constitutional homeopathic treatment according to the principles of classical homeopathy accompanying or following conventional cancer treatment. This consisted in an inpatient stay of approximately one to two weeks for the purpose of finding the correct remedy and phone consultations after patients had gone back home. Details of the treatment have been published elsewhere [12,13].

Our protocol stipulated that patients from both the conventional and homeopathic cohort were to be compared based on the matching criteria of demographic data, clinical data of tumour disease, staging and previous treatment. This entailed that for this direct comparison only patients in a palliative stage could be selected, while in the observational study part all cancer patients - in adjuvant and in palliative stages - who gave their informed consent were included.

Thus, there were three parts to the whole project:

1. A cross-sectional study comparing patient characteristics of the two cohorts at the time of study entry [22].

2. A longitudinal observation of two cohorts over 12 months, one of homeopathic care, one of conventional care with the questions:

a. Is there any difference between the cohorts concerning their conventional or complementary treatment over the course of the year?

b. Are there any changes under the course of the treatment in each cohort related to Qol, psychological wellbeing, fatigue and patient satisfaction?

3. An integrated nested matched pairs comparison between comparable patients in both cohorts regarding their QoL as a feasibility study.

This paper reports on the second part of the project and summarizes the results of the first and the third part.

Measures were patient self-reports, taken at study entry and every 3 months over the course of one year, filled in by patients at intake and sent by post and directly back to the study centre thereafter. Medical records were taken by the treating physicians using case report forms (CRF). Patient records (CRFs) were checked for completeness and information regarding previous treatments and diagnostic information verified at study entry and completion by a monitor. All measures were used in the appropriate and validated German language versions.

Our primary outcome was change in QoL, as measured by the Functional Assessment of Cancer Therapy - General (FACT-G) [23] in conjunction with the Functional Assessment of Chronic Illness Therapy - Spiritual Well-Being (FACIT-Sp) [24]. We defined change scores after 3 months and after 12 months as the points of interest to document short and mid-term effects.

Secondary parameters were:

- Change of fatigue, measured by the Multidimensional Fatigue Inventory, MFI [25].

- Change of psychological wellbeing, measured by the Hospital Anxiety and Depression Scale, HADS [26].

- Patient satisfaction measured by three single items.

Case Report forms documented the sociodemographic parameters, diagnostic information (tumour entity, status, histology, staging, time since diagnosis, progression, metastases), previous treatment (surgery, radiation, chemotherapy, hormone therapy, other treatments), current treatment and survival status.

We included all patients older than 18 years who suffered from a verified tumour disease and who gave informed consent to participation. Since we wanted these data to be as representative as possible we did not apply any exclusion criteria.

Matched Pairs:

Patients who fulfilled the following criteria were included in the matched pairs analysis:

- Histological evidence of malignity

- Evidence for a progressed malignity that is uncurable

- Likely life expectancy of 3 months or more

For each prospective matched-pairs patient all potential matching criteria (see Table 1) were entered in a database and a case vignette was constructed with all relevant data. These were presented to three oncologists otherwise not involved in the treatment of the patients at any time and blind against outcome and further development. Each oncologist decided which patients could be paired. In a final conference they had to find a consensus.

**Table 1:** Matching Criteria

**1. Demographics**
• age • sex • general wellbeing (ECOG-status) • Body Mass Index (BMI)

**2. Tumour Disease**
• type • histology • staging (TNM status) • time of first diagnosis • time of diagnosis of tumour progress • tumour recurrence or metastases and localisation

**3. Previous Therapies**
• surgery • chemotherapy • radiation therapy • hormone therapy • immunotherapy • other therapies

### Data Treatment and Statistics

All case report forms were monitored and information verified against documentation and patient records. Patient self-report data were entered using a scanning system. Data are presented descriptively, with t-tests for dependent data for significant changes within the groups. Effect sizes are expressed as mean differences, using pooled standard deviations in the denominator.

A previous retrospective pilot-study had shown that we can expect a good patient participation in the homeopathic clinic with roughly 200 patients in two years. However, we had no indication of a prospective effect size to go by and hence opted for a feasible number of 200 homeopathy patients recruited over a two year period. We aimed at a core of at least 40 matched pairs and hence at a recruitment of 800 to 1000 patients from the conventional clinics, a figure mentioned as realistic by the participating recruitment centres in several planning meetings. The study was conducted according to Good Clinical Practice (GCP) and the declaration of Helsinki. It was approved by the ethics committee of the University Hospital Freiburg, Germany and the respective local committees of Bellinzona, Switzerland and Stuttgart, Germany.

## Results

**Figure 1:**
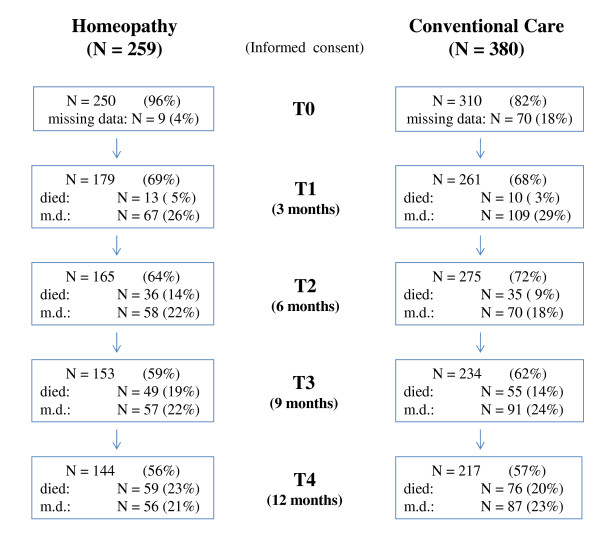
**Flow chart**.

Between 1st Oct 2004 and 30^th ^April 2007 we enrolled 639 patients in the study, 259 from the homeopathic clinics and 380 from the conventional clinics (see Figure 1). Thus, we met our target in the homeopathic clinics but failed by a wide margin to recruit enough patients from the conventional clinics.

Nearly all patients (96%) who had given consent in the homeopathy group (HG) and 82% of all patients included in the conventional group (CG) sent back the questionnaires at the beginning. After 3 months we received back questionnaires from 69% of the HG and 68% of the CG and after 12 months from 56% of the HG and 57% of the CG. In the HG 23% and in the CG 20% of the patients had died. Thus 21% or 23% of all data was missing. Baseline data with exact descriptions of both therapy groups sociodemographics and clinical variables as type of cancer, tumor stages and course of treatment before study entry have been extensively reported elsewhere [22] and are summarised here.

### Differences between the two cohorts at study entry

Patients in the two groups differed in several sociodemographic and disease variables. Homeopathy patients were younger (54 vs. 60 years), had a much higher level of post-16 education (post secondary school/A-level, 54% vs. 25%), and were more likely to be white collar workers or in self-employed jobs (workers, employees 48% vs. 75%).

In both groups the most frequent tumour diagnosis was breast cancer (32% HG vs. 37% CG). In CG more patients with colorectal cancer were found (15% vs. 7%), while more patients with prostate cancer (7% vs. 3%) or melanoma (5% vs. 1%) sought the complementary homeopathic treatment. Patients from the HG were more likely to have a more severe diagnosis or progressed tumour stage (stage I-III only 30% vs. 43% in CG). Homeopathy patients also had a longer elapsed time since their first diagnosis (10 months vs. 3 months), and were more likely to have already had some previous cancer treatment (50% chemotherapy vs. 33%). This confirms the general impression of homeopathic doctors that patients decide to come for homeopathic treatment after having spent some time in the conventional medical system, whereas patients in the CG were more likely to not have tried any other treatment previously.

### Differences between the cohorts concerning therapies during the observation period

As expected, a larger proportion of patients under conventional treatment received chemotherapy or radiation during the 1 year observation period (Table 2). Other treatments, such as immunotherapy or kinase inhibitors were roughly comparable between the groups. Only a few patients, 6,6%, in the CG, did not receive any conventional treatment, whereas 25,6% in the HG had no such treatment, mainly because there was no indication for an antitumour treatment (e.g. adjuvant chemotherapy and/or radiotherapy was already finished before study entry). However, as many as 10% of the HG had an indication for treatment from an oncological point of view but had refused it.

**Table 2:** Conventional Treatment

	HOMEOPATHY N (%)	CONVENTIONAL CARE N(%)
**Therapies t0-t1**		
Surgery	11 (4,3%)	14 (3,7%)
Chemotherapy	53 (20,5%)	244 (64,2%)
Radiation	21 (8,1%)	38 (10,0%)
Hormone therapy	34 (13,2%)	48 (12,6%)
Other therapies (kinase inhibitors, etc.)	25 (9,7%)	52 (13,7%)
		
**Therapies t1-t4**		
Surgery	14 (5,4%)	19 (5,0%)
Chemotherapy 1st line	56 (21,7%)	199 (52,4%)
Chemotherapy 2nd line	20 (7,8%)	64 (16,8%)
Chemotherapy 3rd line	10 (3,9%)	26 (6,8%)
Chemotherapy 4^th ^line	5 (1,9%)	3 (0,8%)
Radiation	22 (8,5%)	57 (15,0%)
Hormone therapy	40 (15,5%)	71 (18,7%)
Other therapies (kinase inhibitors, etc.)	31 (12%)	44 (11,5%)

Patients in both cohorts used other CAM therapies. While in the CG vitamins and mistletoe treatments were used increasingly, patients under homeopathy remained constant or even reduced their usage of these and other CAM treatments (data not shown).

### Changes in Quality of Life, Fatigue and psychological wellbeing

Although patients in the two cohorts were quite different, quality of life (QoL) scores, anxiety, depression and fatigue were very similar in both groups at the beginning of the study. Over the course of 1 year and under homeopathic treatment, QoL improved by a significant degree from a mean of 75.6 to 84.1 in the FACT-G, and from 32.1 to 35.2 in the FACIT-Sp (see Table 3). This is an improvement by an effect size of d = 0.57 for the FACT-G and d = 0.37 for the FACIT-Sp. For patients under conventional care QoL remained largely constant with 75.3 at intake and 78.9 after one year for the FACT-G and 31.0 at intake and 31.6 after a year for the FACIT-Sp. Associated effect sizes are d = 0.2 and d = 0.06. Effects after three months of treatment were similar.

**Table 3:** Quality of Life, Spiritual Wellbeing, Fatigue, Anxiety and Depression

	HOMEOPATHY			CONVENTIONAL CARE		
	**t0**^**1**^	t1 (n = 179)	t4 (n = 140)	**t0**^**1**^	t1 (n = 261)	t4 (n = 191)
**FACT-G**						
**FACT-G**	**75.6 (14.6)**	81.1 (16.9)***	84.1 (15.5)***	**75.3 (17.3)**	76.6 (16.6)*	78.9 (18.1)***
Physical Wellbeing	20.6 (5.9)	22.1 (6.3)***	23.4 (5.1)***	20.6 (5.9)	20.1 (6.3)	21.8 (5.9)***
Social/Family Wellbeing	21.2 (4.0)	21.8 (4.4)**	21.6 (4.7)	21.0 (4.4)	21.9(4.6)***	21.0 (4.8)
Emotional Wellbeing	17.0 (4.2)	16.6 (4.4)***	19.1 (3.9)***	16.9 (5.1)	17.8(4.6)***	18.0 (4.7)***
Functional Wellbeing	16.8 (5.6)	18.6 (6.0)***	20.0 (5.7)***	16.9 (6.0)	17.1 (5.7)	18.2 (6.2)***

**FACIT-Sp**						
**FACIT-Sp**	**32.1 (8.2)**	34.9 (8.3)***	35.2 (8.6)***	**31.0 (8.9)**	32.1 (8.9)**	**31.6 (9.4)**
Meaning Peace	9.1 (4.6)	9.9 (4.7)***	10.2 (4.5)***	8.2 (4.8)	8.4 (4.9)	8.2 (4.8)
Faith	23.4 (5.5)	25.0 (5.0)***	25.0 (5.4)***	23.4 (5.5)	23.8 (5.6)	23.5 (6.0)

**HADS**						
HADS-A	9.7 (1.6)	9.6 (1.1)	9.7 (1.2)	9.9 (1.4)	9.9 (1.4)	10.1 (1.3)
HADS-D	9.0 (1.7)	8.7 (1.7)	8.8 (1.5)	8.3 (1.6)	8.4 (1.8)	8.4 (1.6)

**MFI**						
General Fatigue	11.9 (2.6)	11.4 (2.6)**	11.1 (2.6)**	11.9 (3.2)	12.0 (2.7)	11.8 (2.7)
Physical Fatigue	11.9 (5.2)	10.4 (5.2)***	9.5 (4.9)***	11.6 (5.2)	12.1 (5.2)	10.7 (4.9)**
Reduced Activity	11.8 (4.8)	10.4 (5.0)***	9.5 (3.2)***	11.8 (5.4)	11.5 (5.3)	10.5 (4.9)***
Reduced Motivation	8.8 (3.5)	7.7 (3.9)**	7.4 (3.2)***	9.1 (4.4)	9.0 (4.0)	8.7 (3.7)*
Mental Fatigue	10.6 (4.6)	9.3 (4.7)***	8.3 (4.0)***	9.8 (5.0)	9.3 (4.7)*	9.8 (4.8)

FACT: Functional Assessment of Cancer Therapy; G: General;

FACIT-Sp: Functional Assessment of Chronic Illness Therapy- Spiritual Wellbeing

HADS: Hospital Anxiety and Depression Scale: A - Anxiety; D - Depression

MFI: Multidimensional Fatigue Inventory

Mean (SD)

^1 ^baseline data of patients with valuable data at t1

*P ≤ 0.05; ** p ≤ 0.01; *** p ≤ 0.001

In the homeopathy cohort, but not in the conventional cohort, fatigue decreased significantly in all scales of the Multidimensional Fatigue Inventory (MFI) after three months as well as after one year, but only for mental fatigue, physical activity and physical fatigue did the change amount to half a standard deviation. No changes were seen in both cohorts regarding the HADS.

The data for the sub-cohorts of patients in progressed tumour stages who were eligible for matching were very similar. Here we show only the data for the primary outcome parameter (Table 4). There were no differences between HG and CG in patient satisfaction regarding doctors as well as treatment results (data not shown).

**Table 4:** Quality of Life and Spiritual Wellbeing in palliative patients

	HOMEOPATHY			CONVENTIONAL CARE		
	**t0**^**1**^	t1 (n = 73)	t4 (n = 49)	**t0**^**1**^	t1 (n = 140)	t4 (n = 85)
**FACT-G**	74.6 (15.2)	79.3 (17.3)**	81.9 (15.8)***	73.3 (17.3)	74.8 (17.9)	73.1 (19.2)
**FACIT-Sp**	31.3 (8.8)	34.3 (8.8)***	35.1 (8.8)***	30.6 (9.8)	31.6 (9.2)*	30.1 (9.9)

FACT: Functional Assessment of Cancer Therapy; G: General;

FACIT-Sp: Functional Assessment of Chronic Illness Therapy - Spiritual Wellbeing

Mean (SD)

^1 ^baseline data of patients with valuable data at t1

*P ≤ 0.05; ** p ≤ 0.01; *** p ≤ 0.001

### Matched Pairs

120 patients of *HG *and 206 patients of *CG *met our criteria for the matched-pairs selection. Due to the large differences between the two patient populations, however, only 11 matched pairs could be formed, including 2 pairs each with breast cancer, ovarian cancer, NSCLC, pancreatic- and colon cancer and one pair with glioblastoma. This is not a sufficient number for a reliable comparison. Data described in detail will be submitted separately.

## Discussion

This is, to our knowledge, the first longitudinal study of cancer patients under homeopathic care in a parallel group design with conventional care and the attempt for a nested matched pairs comparison. Our primary aim was to see whether cancer patients under homeopathic care experience a benefit in their quality of life, psychological well-being and fatigue.

At study entry homeopathic patients were, roughly speaking, more severely affected and initiated homeopathic treatment at a later stage than their conventional counterparts. While conventional patients accessed treatment on average 3 months after first diagnosis or after diagnosis of tumour progress, patients in homeopathic care only started treatment 10 months after first diagnosis in an adjuvant situation resp. 7 months after a progress had been diagnosed. This explains the higher rate of patients pre-treated with chemotherapy or radiotherapy in homeopathic care.

While most patients used homeopathic care complementary to an appropriate oncological treatment, 10% refused to have such a treatment for various reasons and seek homeopathic treatment as an alternative. It is important to emphasise at this point that this patient decision was neither encouraged nor discouraged by the homeopathic physicians and has for the most part been taken before patients came to the clinic. All patients had been informed about the fact that the decision as to which therapy to have or not to have falls within their and their doctors' joint responsibility, as there was no experimental treatment within this observational study.

Despite the considerable difference in disease status of the two cohorts it is remarkable that their initial scores in virtually all self-reported measures in quality of life, fatigue, anxiety and depression at baseline are quite comparable. Compared with norm data [27] and oncological cohorts [26,28] our patients have a more severely reduced QoL, more anxiety and depression and comparable fatigue.

During homeopathic care we saw a significant and stable improvement in QoL which, as measured by the FACT G, is sizeable at more than half a standard deviation. We do not see a comparable increase in QoL in the conventionally treated cohort. Such an effect size of more than half a standard deviation is by all standards a clinically relevant improvement [29,30]. Some authors consider an improvement of 3 to 7 points on the FACT-G as the minimally important difference (MID) [31,32], which is achieved by our homeopathy cohort who experienced an improvement by 5.5 points after 3 months and by 8.5 points after 12 months. While depression and anxiety did not change much, as measured by the HADS, fatigue improved significantly across all scales. Homeopathic care patients experienced an improvement of at least half a standard deviation after 12 months for mental fatigue, and both mental and physical fatigue improved to a degree that according to new norm data can be deemed a minimal clinically important difference [28].

In the conventionally treated group improvements were much smaller, failing half a standard deviation change by a wide margin. The MID is marginally reached with an improvement of 3.6 on the FACT-G after 12 months of treatment. Nevertheless, patients of both groups were satisfied with their treatment and their doctors.

One possible explanation for the lack of improvement in QoL in the CG is that considerably more patients of this cohort got chemo- or radiotherapy with possible acute side effects. This accounts for differences in the first three months, but after a time period of twelve months these differences should have washed out, especially because there were even more patients in a palliative treatment situation in the HG, and one important aim of the antitumour therapy is an improvement in QoL in the long run.

Since the cohorts were quite different, as expected from the outset, we refrained from any formal testing of the between group differences for the whole cohorts. For that reason we had anticipated a matched-pairs analysis. Since recruitment in the conventional centres fell considerably below the anticipated numbers we could not obtain the 40 matched pairs anticipated. Also, the complex matching process devised, with 3 oncologists having to agree on a comparatively large set of initial data, led to the fact that only very few potentially matchable pairs could be found. One might consider a randomised study whereby studying homeopathy as a complementary add-on an alternative. However, since there are so many differentiating factors influencing prognosis in tumour therapy, only a very large randomised study or a study using intricate balancing procedures [33] would have a chance of offering valid answers. In view of the experiences of other researchers mentioned in the Introduction and from the experience of our own study we doubt that cancer patients with a vested interest in homeopathic treatment will be willing to be randomised or allocated to treatments by processes other than their decision. It is unlikely that enough patients without preference would be willing to consent to be potentially randomised to either treatment.

A matched pairs study with sufficient power would have to document a number of conventionally treated patients by the factor 10 to 15 more than our study. This is not impossible to achieve, but a considerable effort. While it has been comparatively easy to include enough homeopathically treated patients it is difficult to recruit conventionally treated patients, as they and their physicians lack incentive.

The drawback of this study, that only the observational study part is evaluable by a very small number of comparable pairs, is obvious and does not allow for a final conclusion. The study also has clear strengths: We have subjected all data to rigorous validation procedures and have taken care to verify especially diagnostic and therapeutic information. Patient data are independent and hence likely free from bias. All patients willing to participate have been included, making our sample fairly representative for cancer patients seeking homeopathic care or modern standard conventional care. We have paid attention to comparing only strong exemplars of the treatments in question. The homeopathic clinics studied are well recognised in the field as the absolute experts in homeopathic care in cancer patients and have a very good reputation. So do the conventional clinics representing the state of the art in German oncology.

It is important to notice that we have not studied the effect of homeopathic remedies, but of homeopathic care. This comprises the whole setting of case taking, individualisation, finding the right remedy and following up on the perceived effects in multiple cycles of feedback and adjustment. It goes without saying that this is an intensive communicative, interactive process that operates via many different pathways, some of which are likely to be psychological and very general in the sense of a meaning response [34], some of which might be specific to homeopathic therapy and its usage of the remedies. It is also a likely scenario that homeopathic remedies are only active in an unbroken therapeutic context and that, at least for practical therapeutic reasons, the question whether homeopathic remedies are placebo or not, is irrelevant.

## Conclusions

We have shown that under homeopathic care sizeable benefits were achieved for patients' QoL, as measured by FACT-G and also for spiritual well-being as measured by the FACIT-Sp. The improvement was clinically relevant and statistically significant. It could also be seen in symptoms of physical and mental fatigue. Thus our data suggest that classical homeopathic care could complement conventional cancer care to the benefit of patients. However, the attempt to prove a definite verification by using a Matched Pair control concept did not succeed.

## Competing interests

Harald Walach's position is funded by Heel pharmaceutical company, Baden-Baden, Germany.

None of the other authors has any conflict of interest.

## Authors' contributions

HW, MR, HHB and CG designed the study. JN, MR, LG, and CG collected the data. MR, LG and HHB lead the matching process. CG carried out the statistical analyses. MR, HW, CG, and HHB drafted the manuscript, and all authors participated in the interpretation of the findings, reviewed the manuscript and approved the final manuscript.

## Pre-publication history

The pre-publication history for this paper can be accessed here:

http://www.biomedcentral.com/1471-2407/11/19/prepub
